# Aerobic Fitness Is the Main Determinant of Match-Related Physical Performance Decline in Professional Football Players (Part 2)

**DOI:** 10.1519/JSC.0000000000005546

**Published:** 2026-07-07

**Authors:** Leo Antenucci, Christian Schmidt, Davide Malatesta

**Affiliations:** 1 Institute of Sport Sciences, Biomechanics & Integrative Physiology of Exercise Dynamics (BIPED) laboratory, University of Lausanne, Lausanne, Switzerland; and; 2 Performance Laboratory Football Club Lucerne, Lucerne, Switzerland

**Keywords:** acceleration, deceleration, GNSS tracking, intensity zones, physical fitness parameters, running distance

## Abstract

Antenucci, L, Schmidt, C, and Malatesta, D. Aerobic fitness is the main determinant of match-related physical performance decline in professional football players (part 2). *J Strength Cond Res* 40(11): e1190–e1198, 2026—High-intensity efforts are critical in football performance and declines in running distance and acceleration (ACC) and deceleration (DEC) capacity are often observed after the 60th or 75th minute, partly indicating player fatigue. This study investigated the decline in physical performance during a professional football match and its relationship with physical fitness parameters. Global navigation satellite system data from an entire Swiss first-division season (30 matches) were analyzed for 19 professional football players. The players' speed at the first and second lactate thresholds (sLT_1_/sLT_2_) and peak incremental speed (PIS) were assessed using a maximal incremental treadmill test. The maximal sprint speed (MSS) was determined from match data to calculate the anaerobic speed reserve (ASR = MSS − PIS). The distance covered in low-/high-/very high-speed running (LSR/HSR/VHSR) and sprinting using individual thresholds (<sLT2, sLT2-PIS, PIS-30% ASR, >30% ASR), and the number of ACCs/DECs performed in 4 intensity zones (ACC_1/2/3/4_/DEC_1/2/3/4_: ±0.5–1.0, 1.0–2.0, 2.0–3.0, >3 m·s^−2^) were measured across 6 periods (P1-P6) of 15 minutes of matches. The vertical peak power was measured using squat and countermovement jumps. Significant (*p* ≤ 0.05) declines (4–27%) were observed after the 60th minute across all running, ACC/DEC intensity zones, and in total distance (TD) and total number of ACCs/DECs (ACC_TOT_/DEC_TOT_) counts, except for sprint distance and ACC_1_/DEC_1_. Moderate-to-large correlations were found between aerobic fitness (sLTs and PIS) and decreases in TD, ACC_TOT_/DEC_TOT_, and high-intensity running, ACCs/DECs between P1 (0–15 minutes) and P5 (60–75 minutes) (*r* = 0.46–0.67; *p* ≤ 0.049). Coaches should focus on enhancing players' aerobic fitness to improve their ability to maintain physical performance throughout match-play.

## Introduction

Football is a sport with significant physiologic and cardiovascular demands, where players cover 10–12 km per match (41). Approximately 10–17% of this distance is covered at high-intensity running (HIR: about >14.4 km·h^−1^), with 4–8% at very high-intensity running (VHIR: about >19.8 km·h^−1^) and only 1–3% as sprinting (about >25.2 km·h^−1^), depending on players' age, league level, playing style, and the speed thresholds used (20,41). The total distance (TD) and HIR distance covered during a match are associated with maximal oxygen uptake (V̇o_2_max), maximal aerobic speed (MAS), and lactate/ventilatory thresholds (LT and VT, respectively) in both young (*r* = 0.45–0.77) (5,38) and professional players (*r* = 0.54–0.83) (2,5). Moreover, players perform more than 2000 meters of accelerations (ACC) and decelerations (DEC) per match, of which approximately 10% are high-intensity actions in U-21 football (1,45). Therefore, aerobic fitness is pivotal in sustaining high match intensity, although anaerobic qualities such as maximal strength, power, and rate of force development remain crucial for maximal sprint speed (MSS) and for the ability to perform changes of direction, ACCs, DECs, and jumps that are key at high level (14,48). Finally, although TD covered in matches has slightly decreased since the early 2000s, HIR, VHIR, and sprint distance have increased, highlighting the growing importance of high-intensity efforts in modern football performance (41).

For these reasons, assessing the physiologic and metabolic demand of matches, including high-intensity efforts such as HIR, VHIR, sprinting, and ACCs/DECs, is essential. Several studies have shown a decrease in running distance at various intensities (1,16,30,32,45), and in the number of ACCs and DECs (1,45) during the second half and in the last 15–30-minutes of matches using arbitrary (ARB) thresholds. Studies using individualized (IND) thresholds also reported a second-half decline in HIR distance covered in both professional (9) and junior players (29), but none examined the 5- or 15-minute fluctuations with this method. Nevertheless, compared with ARB, IND speed thresholds are recommended to better capture each player's relative physical load (4,5,20). In contrast, for ACCs and DECs, our previous findings showed that ARB thresholds may be more relevant and accurate than the IND method for assessing these actions during match-play (4). Although IND methods (15,46), data-driven or clustering approaches have been proposed (39), all of these methods require extensive processing of raw global navigation satellite system (GNSS) data, which are not yet implemented in applied monitoring systems. Consequently, the use of conventional ARB thresholds remains the most reproducible and easily applicable solution in professional environments for coaching staff. Therefore, this approach was retained for both ACC and DEC analyses in this study.

To the best of our knowledge, no study has simultaneously analyzed the within-match decline in distances covered across different intensity zones using the IND method and the decline in the number of ACCs and DECs using ARB thresholds in professional football players. Furthermore, only a few studies investigated the relationship between fitness parameters and the decline in match-related physical performance in football, and none of them reported significant correlations (5). Importantly, the studies included in this systematic review only analyzed between halves decline in running performance, potentially overlooking within-match fluctuations (5). However, greater oxidative muscle capacity has been associated with a smaller decline in HIR between halves (33), whereas players with a higher proportion of fast-twitch fibers showed greater reductions in HIR (54). These findings indirectly suggest the likely pivotal role of aerobic fitness in minimizing the decrease in match-related physical performance. Yet no studies have directly explored the relationship between this decline and physical fitness parameters in professional football players.

Therefore, our study aims to investigate (a) the match-related physical performance decline across six 15-minute periods using IND thresholds for running speed and ARB thresholds for ACCs and DECs; (b) the association between players' physical fitness parameters and this decline in professional players for an entire season. We hypothesized (a) a decline in HIR distance covered and in the number of high-intensity ACCs and DECs performed in the last 30 minutes of the match; and (b) these decreases would be associated with the players' aerobic fitness.

## Methods

### Experimental Approach to the Problem

This study used a single-cohort, observational, repeated-measures design aimed at investigating the decline in match-related physical performance and its associations with aerobic fitness parameters. The match-related physical performance was recorded with a GNSS technology at 10 Hz placed in a pouch of a vest worn at the upper back under the jersey (APEX Pro Series Units, STATSports, Northern Ireland), which was previously validated (6,24). The mean number of satellites connected during data collection (mean ± *SD* = 17.6 ± 1.3, range: 14–21) was in line with previous research (3,4,7). After each match, the data were downloaded using the Sonra software (Sonra 4.0, STATSports), converted and exported to an Excel file for analysis.

### Subjects

The 2022–2023 season of a Swiss Super League team (first division) consisting of 33 players who participated in 1 to 2 matches per week, and 4 to 5 trainings were analyzed. Data from 27 Championship and 3 National Cup matches were examined. Three inclusion criteria were established: starting players who played for at least 75 minutes, a minimum of 2 matches, no injury during the game, and matches lasting 90 minutes with 11 players on each side (no red card during the match). The additional time in each half-time and extra-time during cup matches were not considered. No specific intervention was required for this study, which was performed according to the Declaration of Helsinki and for which each player signed a written consent to be included in this study. Authorization by the local Ethics Committee, the CER-VD, was not required for this study, which was performed according to the Declaration of Helsinki and for which each player signed a written consent to be included in this study.

### Procedures

#### Assessment of Match-Related Physical Performance and Laboratory Fitness Testing

The physical performance of the players during matches was evaluated based on the running distance covered and the number of ACCs/DECs performed at 4 intensity zones. The running speed intensities were as follows: low-speed running (LSR), high-speed running (HSR), very high-speed running (VHSR), and sprint distance (see “Intensity zones” for more details), as well as HIR (HSR + VHSR + sprint distance) and VHIR (VHSR + sprint distance) (4). The ACC and DEC intensity zones were ACC_1/2/3/4_ and DEC_1/2/3/4_ (4), respectively (see “Intensity zones” for more details) and >ACC_3_ and >DEC_3_. The TD, the distance covered in the 4 running speed intensities, the total number of ACCs/DECs (ACC_TOT_/DEC_TOT_), and the number of ACCs/DECs performed in the 4 intensity zones were assessed for each of six 15-minute periods to examine the physical performance evolution during a match: P1 = 0–15 minutes; P2 = 15–30 minutes; P3 = 30–45 minutes; P4 = 45–60 minutes; P5 = 60–75 minutes; P6 = 75–90 minutes.

A laboratory discontinuous incremental treadmill test to exhaustion was performed twice by each player to evaluate their aerobic fitness [running speeds associated with lactate thresholds (sLTs) and peak incremental speed (PIS)] and define the IND thresholds used for the first half of the season and readapt them at mid-season for the second part of the season (4). The first test was conducted during the preseason (June–July), and the second test was conducted during the winter break (mid-season: January). In addition, each player also participated in vertical jump tests once during the preseason (June–July), on nonfatiguing days according to team logistics and scheduling constraints.

#### Intensity Zones

The ARB ACC and DEC zones (17) and the IND speed thresholds (20) were obtained based on previous studies and validated in our specific experimental situation (4) (Table 1). The duration of each ACC and DEC must exceed 0.5 seconds to be counted in each intensity zone. The speed zones were established using the results of an incremental test to exhaustion (see the next section), and MSS was assessed in matches as previously described (4) because the club does not include sprint tests in their pre- or in-season protocols to prevent injury risk, and because values can be higher during matches than during a 40-m sprint test (27). Afterward, the anaerobic speed reserve (ASR = MSS − PIS) (29) was calculated to define the upper and lower limits (30% ASR) of VHSR and sprint, respectively (20). The intensity zones were readjusted during the winter break to assess the players' physical performance during matches of the second part of the season.

**Table 1:** Mean individual (IND) speed running thresholds and arbitrary (ARB) acceleration (ACC) and deceleration (DEC) thresholds.*

	LSR; ACC_1_/DEC_1_	HSR; ACC_2_/DEC_2_	VHSR; ACC_3_/DEC_3_	Sprint; ACC_4_/DEC_4_
Speed (km·h^−1^)	<14.9	14.9–18.2	18.2–22.9	22.9–33.9
	< sLT_2_	sLT2—PIS	PIS—30% ASR	30% ASR—MSS
ACCs (m·s^−2^)	0.5–1.0	1.0–2.0	2.0–3.0	>3.0
DECs (−m·s^−2^)	0.5–1.0	1.0–2.0	2.0–3.0	<3.0

* LSR = low-speed running; HSR = high-speed running; VHSR = very high-speed running; ACC_1_/DEC_1_ = acceleration/deceleration in zone 1; ACC_2_/DEC_2_ = acceleration/deceleration in zone 2; ACC_3_/DEC_3_ = acceleration/deceleration in zone 3; ACC_4_/DEC_4_ = acceleration/deceleration in zone 4; sLT_2_ = speed associated with the second lactate threshold; PIS = peak incremental speed; ASR = anaerobic sprint reserve; MSS = maximal sprint speed.

#### Physical Fitness Tests

As previously described (4), a discontinuous incremental treadmill test was performed to evaluate aerobic fitness. Gas exchange was not measured during this test, and no V̇o_2_-related variables (e.g., V̇o_2_max or VTs) were assessed. This test began at a running speed of 7.2 km·h^−1^ with a 1.0% incline, and the speed was progressively increased by 1.8 km·h^−1^ every 3-minute stage. This test is conducted in accordance with SwissOlympic guidelines (50). After each step, players were given a 30-s rest period for lactate sampling from the earlobe (Biosen C-Line, EKF Diagnostic, Barleben, Germany). The speeds associated with the first and second lactate thresholds (sLT_1_ and sLT_2_, respectively) were defined according to Bourdon (11). Peak incremental speed was defined as the running speed of the last fully completed step (S_last, completed, step_) plus the fraction of time spent in the following uncompleted step (α) multiplied by the running speed increment (Δs = 0.01 km·h^−1^) (23): PIS = S_last, completed, step_ + αΔs.

The vertical ground force reactions of 3 countermovement jumps (CMJ) and 3 squat jumps (SJ) were recorded using a twin force plate (Kistler, model Kijump type 9229A, Switzerland) with 2 minutes of recovery between each jump and were automatically handled by the manufacturer's software (MARS, Kistler, Winterthur, Switzerland). The instantaneous vertical power was calculated as the product of the instantaneous vertical ground reaction force (N) and the instantaneous vertical velocity of the center of mass of the body (m·s^−1^). The peak vertical power, which is the peak value during the push-off phase, was collected for each CMJ and SJ of each player. The highest values of the 3 CMJs (CMJ_P_) and the 3 SJs (SJ_P_) in W·kg^−1^ were used as indicators of the maximal power output of the players. The vertical peak power is one of the main outcome variables to characterize vertical jump performance compared with the jump height (35).

### Statistical Analyses

The statistical analyses of the evolution of each intensity zone across the 6 periods were conducted using Jamovi software (1). The results are expressed as mean ± *SD*. A linear mixed model (LMM) was used with the intensity zone of speed, ACC, or DEC as the dependent variable (i.e., 1 analysis per intensity zone). The fixed effect was the “period”, and the player was the clustering variable. The “period | player” was selected as the random coefficient, and the post hoc multiple comparisons with Bonferroni adjustment were performed. The intramatch (between-periods) coefficients of variation (CV) were calculated for each player and metric across P1 and P5, and the team mean CV and 95% confidence interval (CI) were then computed. The relationship between physical fitness parameters and the relative decline (%) in physical performance between P5 and P1 (ΔP5-P1) was assessed using Pearson's correlation (*r*). These 2 periods were selected because not all players played in P6, and the most significant difference appeared between these 2 periods. The correlation coefficients had the following magnitudes: negligible (*r* ≤ 0.1), small (0.1 < *r* ≤ 0.3), moderate (0.3 < *r* ≤ 0.5), large (0.5 < *r* ≤ 0.7), very large (0.7 < *r* ≤ 0.9), and extremely large (0.9 < *r* ≤ 1.0) (19). Shapiro–Wilk tests were conducted to examine the normality of each metric (*p* ≥ 0.05) before each correlation. Pearson's correlation was used when normality was confirmed, and the nonparametric Spearman's correlation was applied otherwise. Cohen's *d* effect sizes were calculated for all between-period differences and interpreted as trivial (<0.2), small (0.2–0.6), moderate (0.6–1.2), large (1.2–2.0), or very large (≥2.0). Statistical significance was set at *p* ≤ 0.05.

## Results

### Subjects

The analysis identified 19 players (age: 24.6 ± 5.2 years (18 - 32 years); height: 181.8 ± 5.1 cm; body mass: 76.3 ± 6.3 kg; body mass index: 23.1 ± 1.2 kg·m^−2^) in 30 different matches, who played at least 2 matches for ≥75 minutes (227 data points for P1 to P5; each player had 11.4 ± 8.6 observations per period), and 17 of whom completed the full game (181 data points for P6; each player had 8.6 ± 9.2 observations per period). Concerning the results of the physical fitness tests, 17 players met the inclusion criteria in the first part of the season [sLT_1_: 10.6 ± 1.0 km·h^−1^; sLT_2_: 14.8 ± 0.8 km·h^−1^; PIS: 18.0 ± 0.7 km·h^−1^; maximal heart rate (HR_max_): 193.6 ± 8.5 bpm; maximal blood lactate concentration ([La]_max_): 11.3 ± 1.8 Mmol·L^−1^], and 16 players met the inclusion criteria in the second part of the season (sLT_1_: 11.0 ± 0.6 km·h^−1^; sLT_2_: 15.1 ± 0.8 km·h^−1^; PIS: 18.6 ± 0.7 km·h^−1^; HR_max_: 193.9 ± 7.6 bpm; [La]_max_: 11.2 ± 2.0 Mmol·L^−1^). The jump tests were conducted by all 19 players during the preseason (CMJ_P_: 54.4 ± 5.2 W·kg^−1^; SJ_P_: 52.8 ± 5.3 W·kg^−1^).

### Match-Related Physical Performance Decline

A significant “period” fixed effect was found for running distance, number of ACCs and DECs in each intensity zone, and total values (TD, ACC_TOT_, DEC_TOT_; *p* ≤ 0.032; Table 2).

**Table 2:** Mean ± *SD* distance covered, number of accelerations (ACC) and decelerations (DEC) performed in each intensity zone by period (P).*

Metric	P1	P2	P3	P4	P5	P6	Period effect (*p*)
LSR (m)	1,335 ± 140	1,365 ± 123	1,335 ± 126	**1,285 ± 157** ^**1,2,3**^	**1,278 ± 117** ^**1,2,3**^	**1,267 ± 117** ^**1,2,3**^	<0.001
HSR (m)	199 ± 59	**180 ± 58** ^**1**^	**175 ± 56** ^**1**^	**174 ± 57** ^**1**^	**165 ± 52** ^**1**^	**146 ± 51** ^**1,2,3,4**^	<0.001
HIR (m)	393 ± 113	**350 ± 100** ^**1**^	**357 ± 99** ^**1**^	**354 ± 103** ^**1**^	**329 ± 99** ^**1**^	**296 ± 96** ^**1,2,3,4**^	<0.001
VHSR (m)	136 ± 53	**120 ± 47** ^**1**^	123 ± 45	125 ± 46	**114 ± 44** ^**1**^	**104 ± 45** ^**1**^	<0.001
VHIR (m)	194 ± 75	**170 ± 62** ^**1**^	182 ± 66	180 ± 66	**164 ± 67** ^**1**^	**150 ± 67** ^**1**^	<0.001
Sprint distance (m)	58.2 ± 37.4	49.5 ± 30.5	58.5 ± 35.8	54.8 ± 33.4	50.0 ± 34.6	46.0 ± 34.6	0.029
TD (m)	1728 ± 174	1715 ± 157	1,692 ± 166	**1,639 ± 209** ^**1,2**^	**1,607 ± 158** ^**1,2,3**^	**1,564 ± 159** ^**1,2,3,4**^	<0.001
ACC_1_ (nb)	36.6 ± 7.1	37.7 ± 6.4	36.3 ± 6.2	35.9 ± 6.5	35.8 ± 6.4	36.3 ± 7.3	0.032
ACC_2_ (nb)	44.2 ± 7.8	43.6 ± 8.1	43.1 ± 7.4	**41.6 ± 7.8** ^**1**^	**39.6 ± 6.9** ^**1,2,3**^	**39.9 ± 7.3** ^**1,2,3**^	<0.001
ACC_3_ (nb)	22.3 ± 4.8	**20.4 ± 5.4** ^**1**^	**20.0 ± 5.3** ^**1**^	**20.2 ± 5.1** ^**1**^	**18.0 ± 4.5** ^**1,2,3,4**^	**17.9 ± 4.7** ^**1,2,3,4**^	<0.001
> ACC_3_ (nb)	34.4 ± 6.5	**31.9 ± 7.2** ^**1**^	**30.8 ± 6.8** ^**1**^	**31.5 ± 6.8** ^**1**^	**27.9 ± 5.9** ^**1,2,3,4**^	**27.3 ± 6.0** ^**1,2,3,4**^	<0.001
ACC_4_ (nb)	12.1 ± 3.8	11.5 ± 4.3	10.8 ± 4.0	11.3 ± 4.1	**9.9 ± 3.4** ^**1,2,4**^	**9.5 ± 3.4** ^**1,2,4**^	<0.001
ACC_TOT_ (nb)	115 ± 11	113 ± 12	**110 ± 12** ^**1**^	**109 ± 13** ^**1,2**^	**103 ± 11** ^**1,2,3,4**^	**103 ± 13** ^**1,2,3,4**^	<0.001
DEC_1_ (nb)	37.0 ± 7.3	36.3 ± 7.6	34.8 ± 7.1	35.1 ± 7.2	35.0 ± 7.8	35.5 ± 7.7	0.003
DEC_2_ (nb)	44.8 ± 7.7	43.3 ± 8.1	**41.9 ± 8.0** ^**1**^	**41.1 ± 7.7** ^**1**^	**39.3 ± 7.3** ^**1,2**^	**38.7 ± 7.5** ^**1,2,3**^	<0.001
DEC_3_ (nb)	18.6 ± 4.4	18.4 ± 4.7	17.3 ± 4.4	**16.7 ± 4.6** ^**1,2**^	**15.5 ± 4.1** ^**1,2,3**^	**15.5 ± 4.5** ^**1,2**^	<0.001
> DEC_3_ (nb)	33.1 ± 6.7	31.8 ± 6.7	31.2 ± 6.3	**29.8 ± 7.1** ^**1**^	**27.4 ± 5.8** ^**1,2,3**^	**27.0 ± 6.1** ^**1,2,3**^	<0.001
DEC_4_ (nb)	14.5 ± 4.5	13.4 ± 4.5	13.9 ± 4.5	13.1 ± 4.6	**11.9 ± 4.2** ^**1,2,3**^	**11.5 ± 4.1** ^**1,3**^	<0.001
DEC_TOT_ (nb)	115 ± 11	111 ± 13	**108 ± 13** ^**1**^	**106 ± 13** ^**1,2**^	**102 ± 12** ^**1,2,3,4**^	**101 ± 13** ^**1,2,3,4**^	<0.001

* LSR = low-speed running; HSR = high-speed running; HIR = high-intensity running (HSR + VHSR + sprint distance); VHSR = very high-speed running; VHIR = very high-intensity running (VHSR + sprint distance); ACC1/2/3/4 = accelerations in zones 1, 2, 3, and 4, respectively; >ACC3 = ACC3 + ACC4; ACCTOT = total number of accelerations; DEC1/2/3/4 = decelerations in zones 1, 2, 3, and 4, respectively; >DEC3 = DEC3 + DEC4; DECTOT = total number of decelerations; P1 = period 1: 0 15 min; P2 = period 2: 15 30 min; P3 = period 3: 30 45 min; P4 = period 4: 45 60 min; P5 = period 5: 60 75 min; P6 = period 6: 75 90 min. In bold post hoc significantly lower (*p* ≤ 0.05) than P1 (1), P2 (2), P3 (3), and P4 (4).

The LSR distance covered during P4, P5, and P6 was significantly lower than that during P1 (≥4%; *d* = 0.30, 0.40, and 0.45, respectively; *p* ≤ 0.026), P2 (≥6%; *d* = 0.50, 0.62, and 0.72, respectively; *p* < 0.001), and P3 (≥4%; *d* = 0.34, 0.45, and 0.63, respectively; *p* ≤ 0.024; Table 2). The HSR distance covered during P2, P3, P4, P5, and P6 was significantly lower than that during P1 (≥10%; *d* = 0.33, 0.43, 0.46, 0.62 and 0.79, respectively; *p* ≤ 0.028), and the HSR distance covered during P6 was lower than that covered during P2, P3, and P4 (≥16%; *d* = 0.53, 0.45 and 0.50, respectively; *p* ≤ 0.023; Table 2). The HIR distance covered during P2, P3, P4, P5, and P6 was significantly lower than that during P1 (≥11%; *d* = 0.39, 0.35, 0.34, 0.57 and 0.67, respectively; *p* ≤ 0.030), and the HIR distance covered during P6 was lower than that covered during P2, P3, and P4 (≥15%; *d* = 0.38, 0.54 and 0.49, respectively; *p* ≤ 0.016; Table 2). The distance covered during P2, P5, and P6 was significantly lower than that covered during P1 for VHSR (≥12%; *d* = 0.27, 0.39 and 0.42, respectively; *p* ≤ 0.046; Table 2) and VHIR (≥12%; *d* = 0.30, 0.37 and 0.37, respectively; *p* ≤ 0.036; Table 2). Despite a significant “period” effect also for sprint distance, post hoc analysis revealed no significant difference among periods (*p* ≥ 0.213; Table 2). The TD covered during P4, P5, and P6 was significantly lower than that during P1 (≥5%; *d* = 0.39, 0.67 and 0.71, respectively; *p* ≤ 0.001) and P2 (≥4%; *d* = 0.38, 0.57 and 0.78, respectively; *p* ≤ 0.004). During P5 and P6, TD was significantly lower than that during P3 (≥5%; *d* = 0.50 and 0.85, respectively; *p* ≤ 0.002); during P6, it was significantly reduced compared with that during P4 (5%; *d* = 0.35; *p* = 0.036; Table 2).

Between-periods CV increased with running intensity, indicating that higher metrics showed greater fluctuations. These values and their 95% CI are presented in Table 3.

**Table 3:** Variability of between-periods match physical performance expressed as coefficients of variation (CV) and their 95% confidence intervals (CI).*

Metric	Between-periods CV (CI)
LSR	8.0% (7.3–8.8%)
HSR	21.9% (20.7–23.2%)
HIR	21.4% (19.7–23.0%)
VHSR	29.7% (27.1–32.2%)
VHIR	29.8% (27.5–32.1%)
Sprint distance	58.4% (53.2–63.6%)
TD	8.5% (7.8–9.3%)
ACC_1_	15.3% (14.0–16.7%)
ACC_2_	15.4% (14.0–16.9%)
ACC_3_	22.8% (21.3–24.3%)
> ACC_3_	17.7% (16.3–19.0%)
ACC_4_	29.4% (26.6–32.1%)
ACC_TOT_	9.5% (8.5–10.6%)
DEC_1_	16.6% (15.4–17.7%)
DEC_2_	15.3% (14.0–16.5%)
DEC_3_	23.8% (21.7–26.0%)
> DEC_3_	18.7% (17.0–20.4%)
DEC_4_	26.3% (23.8–28.8%)
DEC_TOT_	9.6% (8.9–10.2%)

* LSR = low-speed running; HSR = high-speed running; HIR = high-intensity running (HSR + VHSR + sprint distance); VHSR = very high-speed running; VHIR = very high-intensity running (VHSR + sprint distance); ACC_1/2/3/4_ = accelerations in zones 1, 2, 3, and 4, respectively; >ACC_3_ = ACC_3_ + ACC_4_; ACC_TOT_ = total number of accelerations; DEC_1/2/3/4_ = decelerations in zones 1, 2, 3, and 4, respectively; >DEC_3_ = DEC_3_ + DEC_4_; DEC_TOT_ = total number of decelerations.

Despite a significant “period” effect for ACC_1_, post hoc analysis revealed no differences among periods (*p* ≥ 0.196; Table 2). The number of ACC_2_ performed during P4, P5, and P6 was significantly lower than that during P1 (≥6%; *d* = 0.27, 0.49 and 0.52, respectively; *p* ≤ 0.045), and the number of ACC_2_ during P5 and P6 was significantly lower than those during P2 (≥8%; *d* = 0.43 and 0.53, respectively; *p* = 0.002 for both) and P3 (≥7%; *d* = 0.42 and 0.48, respectively; *p* = 0.007 for both; Table 2). The number of ACC_3_ performed during P2, P3, P4, P5, and P6 significantly decreased compared with that during P1 (≥9%; *d* = 0.21, 0.37, 0.36, 0.71 and 0.67, respectively; *p* ≤ 0.023), and the number of ACC_3_ during P5 and P6 was significantly lower than those during P2 (12% for both; *d* = 0.39 and 0.42, respectively; *p* ≤ 0.045), P3 (≥10%; *d* = 0.33 and 0.35, respectively; *p* ≤ 0.047) and P4 (11% for both; *d* = 0.39 and 0.38, respectively; *p* ≤ 0.030; Table 2). The number of >ACC_3_ performed during P2, P3, P4, P5, and P6 significantly decreased compared with those during P1 (≥7%; *d* = 0.34, 0.48, 0.43, 0.92 and 0.90, respectively; *p* ≤ 0.017), and the number of >ACC_3_ during P5 and P6 was significantly lower than those during P2 (≥13%; *d* = 0.57 and 0.61, respectively; *p* ≤ 0.004), P3 (≥9%; *d* = 0.42 and 0.50, respectively; *p* ≤ 0.009) and P4 (≥11%; *d* = 0.56 and 0.54, respectively; *p* ≤ 0.003; Table 2). The number of ACC_4_ performed during P5 and P6 was significantly lower than those during P1 (≥18%; *d* = 0.57 and 0.55, respectively; *p* ≤ 0.002), P2 (≥14%; *d* = 0.36 and 0.43, respectively; *p* ≤ 0.018) and P4 (≥13%; *d* = 0.32 and 0.35, respectively; *p* ≤ 0.040; Table 2). The number of ACC_TOT_ performed during P3, P4, P5, and P6 was significantly lower than that during P1 (≥4%; *d* = 0.38, 0.46, 0.85 and 0.82, respectively; *p* ≤ 0.009) and that during P4, P5, and P6 was significantly lower than that during P2 (≥4%; *d* = 0.30, 0.73 and 0.81, respectively; *p* ≤ 0.039). The number of ACC_TOT_ performed during P5 and P6 was significantly lower than those during P3 (6% for both; *d* = 0.50 and 0.60, respectively; *p* ≤ 0.003) and P4 (6% for both; *d* = 0.41 and 0.42, respectively; *p* ≤ 0.046; Table 2). As with running efforts, between-periods CV increased with ACC intensity, and are reported with their 95% IC in Table 3.

Despite a significant “period” effect for DEC_1_, post hoc analysis reveals no difference among periods (*p* ≥ 0.083; Table 2). The number of DEC_2_ performed during P3, P4, P5, and P6 was significantly lower than that during P1 (≥6%; *d* = 0.30, 0.42, 0.59 and 0.68, respectively; *p* ≤ 0.022); DEC_2_ during P5 and P6 was significantly lower than that during P2 (≥9%; *d* = 0.43 and 0.58, respectively; *p* ≤ 0.003). For this zone, the number of DECs performed during P6 was significantly lower than that during P3 (8%; *d* = 0.38; *p* = 0.022; Table 2). The number of DEC_3_ performed during P4, P5, and P6 was significantly lower than those during P1 (≥10%; *d* = 0.32, 0.54 and 0.51, respectively; *p* ≤ 0.046) and P2 (≥9%; *d* = 0.27, 0.48 and 0.49, respectively; *p* ≤ 0.047) and the number of DEC_3_ during P5 was significantly lower than that during P3 (10%; *d* = 0.33; *p* = 0.032; Table 2). The number of >DEC_3_ performed during P4, P5, and P6 was significantly lower than those during P1 (≥10%; *d* = 0.45, 0.79 and 0.68, respectively; *p* ≤ 0.005) and the number of >DEC_3_ during P5 and P6 was significantly lower than that during P2 (≥14%; *d* = 0.59 and 0.56, respectively; *p* ≤ 0.001) and P3 (≥12%; *d* = 0.51 and 0.59, respectively; *p* ≤ 0.003; Table 2). The number of DEC_4_ performed during P5 and P6 was significantly lower than those during P1 (≥18%; *d* = 0.52 and 0.51, respectively; *p* ≤ 0.004) and P3 (≥14%; *d* = 0.38 and 0.50, respectively; *p* ≤ 0.008); DEC_4_ during P5 was lower than that during P2 (11%; *d* = 0.30; *p* = 0.042; Table 2). The number of DEC_TOT_ performed during P3, P4, P5, and P6 was significantly lower than that during P1 (≥6%; *d* = 0.54, 0.67, 0.96 and 0.96, respectively; *p* < 0.001); DEC_TOT_ during P4, P5, and P6 was significantly lower than that during P2 (≥5%; *d* = 0.36, 0.69 and 0.85, respectively; *p* ≤ 0.006). Finally, the number of DEC_TOT_ performed during P5 and P6 was significantly lower than those during P3 (6% for both; *d* = 0.46 and 0.58, respectively; *p* ≤ 0.003) and P4 (6% for both; *d* = 0.31 and 0.39, respectively; *p* ≤ 0.043; Table 2). Similarly to ACC, between-periods CV increased with DEC intensity (Table 3).

### Correlations Between Match-Related Physical Performance Decline and Physical Fitness Parameters

The correlations between the physical fitness tests and the decline in physical performance in match-play were only calculated with the ΔP5–P1 relative drop (%) because not all players played in P6, and the most significant difference appeared between these 2 periods. There were significantly positive large correlations between sLT_1_ and the relative decline in VHSR, in TD covered, in number of ACC_3_ and number of >DEC_3_ performed; between sLT_2_ and the relative decrease in VHSR, TD covered, the number of ACC_3_, > ACC_3_, ACC_TOT_, >DEC_3_ and DEC_4_ performed; and between PIS and the relative decline in VHSR distance covered (Table 4 and Figure 1). There were significant positive moderate correlations between sLT_1_ and the relative decline in HIR distance covered and number of >ACC_3_ performed; between sLT_2_ and the relative decrease in HIR and VHIR distance covered (Table 4). No other significant correlation was detected with other aerobic fitness parameters (*p* ≥ 0.069). There was no significant correlation between vertical peak powers (CMJ_P_ and SJ_P_), and all speed, ACC, or DEC intensity zones (*p* ≥ 0.074).

**Table 4:** Correlation (*r*) between incremental treadmill tests results and relative decline in physical performance between period 1 and period 5 [ΔP5-P1 (%)].*

	sLT_1_	sLT_2_	PIS
ΔP5-P1 (%)	*r*_*(p)*_	*r*_*(p)*_	*r*_*(p)*_
LSR	0.26 _(0.286)_	0.37 _(0.120)_	0.24 _(0.320)_
HSR	0.37 _(0.120)_	0.37 _(0.123)_	0.16 _(0.527)_
HIR	0.46*§_**(0.049)**_	0.48*§_**(0.038)**_	0.31 _(0.205)_
VHSR	0.55*¥_**(0.016)**_	0.63*¥_**(0.004)**_	0.52*¥_**(0.022)**_
VHIR	0.43 _(0.069)_	0.48*§_**(0.036)**_	0.39 _(0.095)_
Sprint distance	−0.07 _(0.777)_	0.092 _(0.709)_	0.122 _(0.619)_
TD	0.54*¥_**(0.017)**_	0.67*¥_**(0.002)**_	0.42 _(0.071)_
ACC_1_	−0.07 _(0.775)_	−0.08 _(0.758)_	0.02 _(0.937)_
ACC_2_	−0.10 _(0.672)_	0.12 _(0.633)_	0.09 _(0.723)_
ACC_3_	0.52*¥_**(0.0.24)**_	0.64*¥_**(0.003)**_	0.28 _(0.242)_
>ACC_3_	0.49*§_**(0.033)**_	0.54*¥_**(0.016)**_	0.28 _(0.251)_
ACC_4_	−0.03 _(0.899)_	−0.17 _(0.493)_	−0.06 _(0.817)_
ACC_TOT_	0.29 _(0.233)_	0.51*¥_**(0.025)**_	0.29 _(0.227)_
DEC_1_	−0.12 _(0.624)_	0.15 _(0.548)_	0.19 _(0.426)_
DEC_2_	−0.01 _(0.983)_	0.06 _(0.815)_	−0.05 _(0.839)_
DEC_3_	0.24 _(0.327)_	0.29 _(0.227)_	0.24 _(0.320)_
>DEC_3_	0.50*¥_**(0.028)**_	0.62*¥_**(0.005)**_	0.42 _(0.071)_
DEC_4_	0.38 _(0.106)_	0.61*¥_**(0.005)**_	0.33 _(0.174)_
DEC_TOT_	0.16 _(0.524)_	0.42 _(0.077)_	0.40 _(0.088)_

* LSR = low-speed running; HSR = high-speed running; HIR = high-intensity running (HSR + VHSR + sprint distance); VHSR = very high-speed running; VHIR = very high-intensity running (VHSR + sprint distance); ACC_1/2/3/4_ = accelerations in zones 1, 2, 3, and 4, respectively; >ACC_3_ = ACC_3_ + ACC_4_; ACC_TOT_ = total number of accelerations; DEC_1/2/3/4_ = decelerations in zones 1, 2, 3, and 4, respectively; >DEC_3_ = DEC_3_ + DEC_4_; DEC_TOT_ = total number of decelerations; sLT_1_ = speed associated with the first lactate threshold; sLT_2_ = speed associated with the second lactate threshold; PIS = peak incremental speed. Significant correlation (*p* ≤ 0.05: *) with moderate (0.3–0.5: §) and high (0.5–0.7: ¥) magnitudes.

In bold post hoc significantly lower (p ≤ 0.05) than P1 (1), P2 (2), P3 (3), and P4 (4).

**Figure 1.**
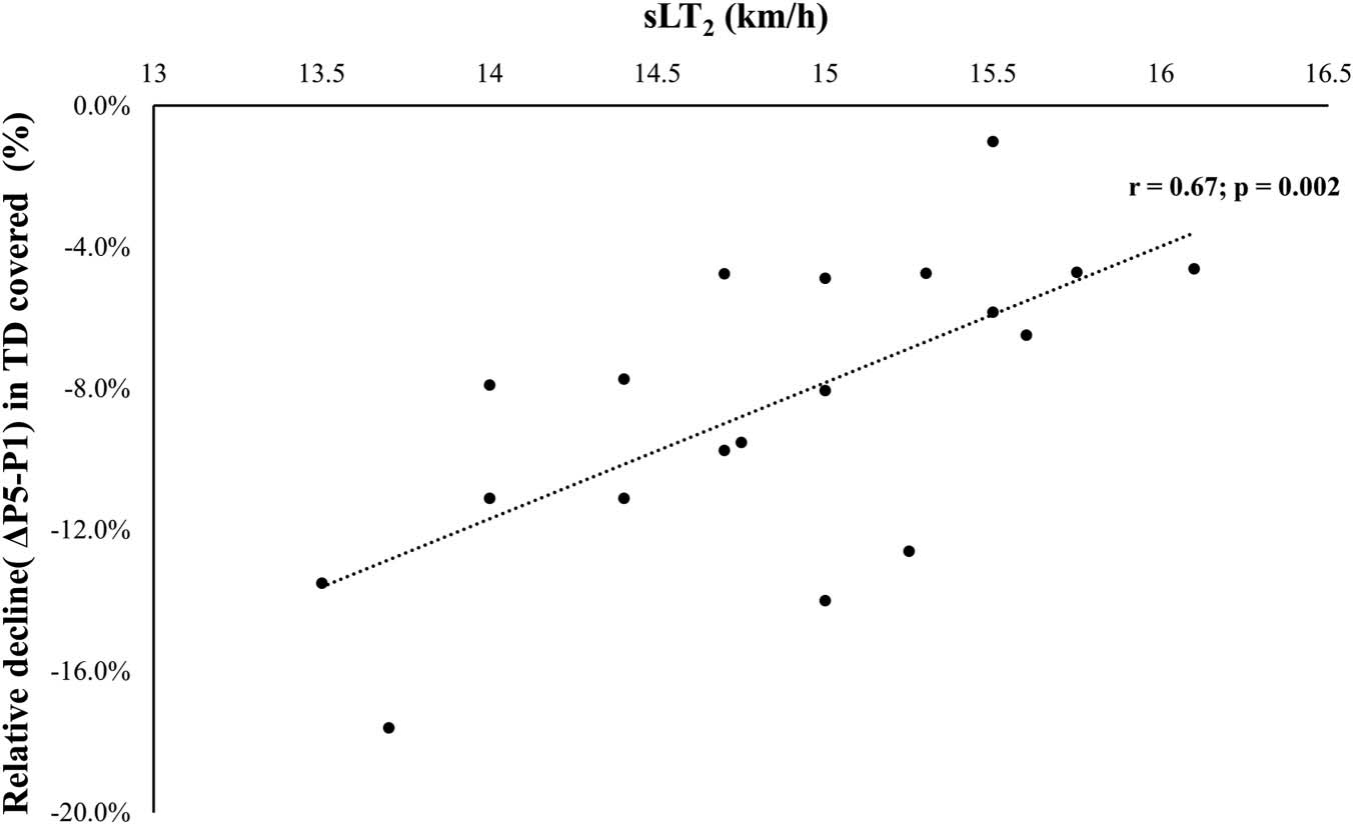
Individual values (*n* = 19) for the mean relative decline between P5 and P1 [ΔP5–P1 (%)] in total distance (TD) covered by the players, plotted against the speed associated with their second lactate threshold (sLT_2_).

## Discussion

This study investigated the decline in match-related physical performance in a professional football team and its associations with aerobic fitness parameters and vertical jump maximal power. The results confirmed our first hypothesis, showing a decrease in physical performance toward the end of the match, with a significant drop in TD and distance covered across all speed-intensity zones, except for sprint distance. A similar pattern was observed across all ACC/DEC intensity zones and for ACC_TOT_/DEC_TOT_, with a significant decline starting from P5. Our second hypothesis was also confirmed. The relative ΔP5–P1 in HIR, VHSR, VHIR, and TD, as well as in the number of ACC_3_, >ACC_3_, ACC_TOT_, >DEC_3_, and DEC_4_, was correlated with aerobic fitness parameters (sLT_1_, sLT_2_, and/or PIS). However, no significant correlations were found for LSR, HSR, or sprint distance. Finally, vertical jump performance was not related to the ability to maintain physical performance throughout the match.

Although our study did not directly compare the 2 halves, the 15-minute period analysis revealed clear reductions in TD, LSR, HSR, as well as in ACC_2/3/4_/DEC_2/3/4_ and ACC_TOT_/DEC_TOT_ (Table 2), indirectly corroborating the well-documented second-half decline in match physical performance (1,9,12,18,32,45). By analyzing within-match changes 15-minute period-by-period, our approach provides a more precise indication of when accumulated fatigue begins to affect performance. Total distance and LSR decreased immediately from the start of the second half (P4 vs. P1–P3: 4–6%), with a progressive reduction toward the end of the match (P6 vs. P1–P3: 5–9%; Table 2). HSR/HIR exhibited a 2-phase decline. An initial drop occurred from P2 (10–11% lower than P1), followed by a relatively stable phase from P2 to P4 (±1–3% and nonsignificant differences across these periods), and then a second reduction from P5 (5–8%, nonsignificant) culminating in a marked and significant decrease in P6 (16–27%; Table 2), consistent with previous descriptions of reductions in the final match phase (32). Regarding VHSR, VHIR, and sprint distance, changes occurred across distinct phases of the match. A first drop was observed in P2 compared with P1 (12–15%), followed by a slight increase in P3–P4 relative to P2 (but still below P1), and then a second, more pronounced and progressive decline occurred in P5 (14–16%) and continued in P6 compared with P2–P4 (21–24%). For sprint distance, however, none of these changes reached statistical significance (Table 2), in line with some studies (12) but contrasting with others (32,45).

Although accumulated fatigue, including mechanisms such as reduced muscle glycogen concentration (8), central and peripheral neuromuscular dysfunction (43), and increased creatine kinase concentrations (17), contributes to physical performance decline, several other factors may also account for the varying magnitudes and timing of decrease across intensities. Methodological differences (e.g., ARB vs. IND thresholds), characteristics of the study population (professional vs. youth players), and whether sprint distance or sprint count is analyzed all influence the detection of changes. Contextual elements such as pacing strategies, match outcome, tactical role, playing position, or match importance may likewise modulate running output (12). For instance, players may intentionally reduce low-intensity activity to maintain their ability to perform high-intensity actions (12), while positional demands differentially affect VHSR declines (30). In addition, the high between-period CV at higher intensities (VHSR/VHIR: 29.7–29.8%), and especially in sprinting (58.4%; Table 3), may partly explain the absence of statistical significance for sprint distance despite clear numerical reductions. Finally, effective playing time is known to decrease in the second half and particularly toward the end of the match, which affects the interpretation of physical performance decline (25,44). When running metrics are adjusted accordingly, TD and LSR tend to increase, whereas VHSR and sprint actions remain stable in the second half (44). Moreover, when comparing P6 with P1, the overall decline in running intensities is more than twice smaller when effective playing time is considered (25). These findings indicate that absolute (unadjusted) declines must be interpreted with caution. Together, these factors provide a coherent explanation for the heterogeneous declines observed across intensity zones in our study.

With respect to ACCs and DECs, except for Zone 1, all intensities and ACC_TOT_/DEC_TOT_ showed a decrease in the number of actions performed in P5 (8–19%) and P6 (8–22%) compared with P1 and P2 or P3 (Table 2). In most cases, significant differences were also observed relative to a third and/or a fourth period (P3 and/or P4, depending on the zone and period; Table 2), confirming an important decline from P5 onward, as previously reported (1,45). In previous studies (1,45), the decline in ACCs and DECs appeared to start in P5 but became more pronounced in P6. The slight difference between our findings (earlier decline in P5) and previous results (P6) may partly be because of methodological differences. Notably, those studies focused on younger teams (U-21) and included nearly half as many matches as the present analysis.

Unlike speed-based metrics, where differences between intensities were observed, our results revealed a relatively homogeneous decline across all ACC/DEC variables, suggesting that muscle fatigue may play a more prominent role in these efforts. However, ACC/DEC metrics also showed lower between-period variability and more stable CVs (≤29.4%) than running speed zones (which reached 58.4%; e.g., CV of VHSR > CV of ACC_3_/DEC_3_; Table 3). This lower variability may contribute to the earlier and more clearly detectable decline (from P5) in ACC/DEC metrics, even at higher intensities. Therefore, it remains difficult to determine whether fatigue affects ACCs/DECs more strongly, or whether running-speed metrics are simply more context dependent, making their decline less visible within and between periods.

According to the systematic review by Aquino et al. (5), only 3 studies investigated the link between the second-half physical performance decline and players' physical fitness, and none reported significant association. In contrast, using 15-minute analysis periods with full-season data from professional players and multiple aerobic parameters, our study showed moderate to large correlations between the relative ΔP5–P1 in running metrics (i.e., TD, HIR, VHSR, and VHIR) and aerobic fitness (sLT_1_, sLT_2_, and PIS; Table 4). In other words, players with higher aerobic fitness exhibited a smaller decline in TD, HIR, VHSR, and VHIR distance covered (Table 4 and Figure 1). However, the decline in LSR and HSR was not associated with aerobic fitness. Although LSR showed significant changes across periods, the relative ΔP5–P1 was much lower (4%) than higher intensities (15–16%), which may partly explain this absence of correlation. In addition, HIR and VHIR have been reported to show stronger associations with postmatch fatigue (17), which may explain their higher correlations with aerobic parameters. Finally, no link was found for sprint distance, likely because of the absence of a significant decline and the very high between-periods CV (Table 3).

Among aerobic indicators, sLT_2_ showed the strongest correlations (*r* = 0.48–0.67; Table 4), and even outperformed PIS. This is likely explained by the fact that PIS often exceeds the velocity associated with V̇o_2_max (13), whereas sLT_2_ reflects an intensity close to or slightly above critical speed/power (21,40), which is one of the best indicators of aerobic fitness (36). Furthermore, sLT_1_ was significantly correlated with TD, HIR, and VHSR and tended to be positively associated with VHIR distance covered (Table 4), suggesting that base endurance level (i.e., foundational level of aerobic fitness) also contributes to limiting the high-intensity efforts decline. From an applied perspective, these findings suggest a potential revision of running speed zones by adding a new IND threshold at sLT_1_, creating a low-intensity zone (<sLT_1_) and a moderate-intensity zone (sLT_1_–sLT_2_). This novel approach enables a more accurate and IND assessment of running demand, given that walking (<7.1 km·h^−1^) and jogging (7.1–14.4 km·h^−1^) are tactically relevant in elite football (31).

Our results corroborate previous findings linking higher oxidative capacity to a smaller second-half decline in TD covered (33), and showing that players with a higher proportion of fast muscle fibers experience larger reductions in distance covered above 15 km·h^−1^ (54). Together, these findings reinforce the importance of aerobic fitness for maintaining performance at the end of matches, when fatigue accumulates and most goals are scored (42). However, as previously explained, because contextual and tactical factors can substantially influence running output (12), and because of the high CVs, the associations found in this study should be interpreted with caution and confirmed by future studies considering playing position, match outcome, match location, and tactical aspects.

Regarding ACCs and DECs, we also found moderate to large correlations between sLT_1_ (*r* = 0.49–0.52) or sLT_2_ (*r* = 0.51–0.64) and the decline in the number of ACC_3_, >ACC_3_, >DEC_3_, DEC_4_, and ACC_TOT_ performed. These findings are in line with those obtained with running distances (Table 4) and confirm that aerobic fitness contributes to sustaining repeated explosive actions across the match. To the best of our knowledge, this is the first study to investigate these associations in professional players, supporting earlier findings showing that players with slower muscle typology exhibit smaller second-half declines in moderate-to-high ACC/DEC actions (54).

No correlation was found between SJ_P_ or CMJ_P_ and the decline in distance covered in speed zones, or in the number of ACCs/DECs at different intensities, indicating that vertical power output is not related to the decline in physical performance during the match. This likely reflects a mismatch between a single maximal effort (vertical jump) and the repeated nature of running, ACCs, and DECs during match-play. Both fast-twitch fiber dominant (10) and sprint-trained athletes (53) typically perform better in repeated jump tests but show larger decrements, suggesting that such tests may better capture the ability to sustain high-intensity efforts under fatigue than a single maximal jump. In addition, because sprint distance did not significantly decline during the match, the lack of association with jump performance is expected. These findings highlight that vertical jump tests, while informative for explosive power, may have limited relevance for predicting match-related performance declines. However, future research should investigate the relationship between repeated maximal efforts, such as jumps or sprints, and the decline in within-match physical performance.

The first limitation of this study is that it was conducted on a single professional team for a single season, whereas some previous studies analyzed multiple teams (29,47) or followed the same squad for several seasons (2). Although the sample included 19 players and a relatively high total number of observations (*n* = 227 for P1–P5; *n* = 181 for P6), the rule change allowing 3 to 5 substitutions per match since 2020 (13) reduced the number of players meeting the inclusion criteria, particularly in some playing positions, contributing to an imbalance in observations across players. However, the use of LMM appropriately handles such unbalanced repeated-measures structures and provides robust, unbiased estimates. Because of these constraints, the sample size within playing positions was insufficient to perform positional analyses or position-specific correlations. Another limitation of this study is that all fitness assessments (incremental test and vertical jump test) were conducted at 2 time points, with the first one during the preseason, when players' physical capacities may still evolve (47). Although changes are generally moderate during this phase with minor or specific changes thereafter (22,28), this timing may have slightly influenced the observed associations. Nevertheless, the preseason and mid-season remain the most practical windows for testing in applied environments, owing to logistical and scheduling constraints (e.g., injuries, transfers), and this approach is widely adopted in professional football. Future studies should confirm these findings across different teams, seasons, and competitive levels, by distinguishing between playing positions and using alternative approaches to assess players' physical fitness. Finally, contextual factors and effective playing time, which may influence both performance decline and its relationship with aerobic fitness, were not considered in this study and should be examined in future research.

Our study revealed that several match-related physical performance parameters declined during final stages of professional football matches. The most affected actions were ACCs and DECs, with a significant decrease from the 60th minute across all intensity zones (except ACC_1_/DEC_1_) and for ACC_TOT_/DEC_TOT_ when using ARB thresholds. With the IND method, TD and LSR distance covered also declined toward the end of the match, whereas HSR, HIR, VHSR, and VHIR showed more heterogeneous patterns, with reductions emerging in P5 and becoming more pronounced in the final 15 minutes. In contrast, sprint distance remained relatively stable throughout the match, suggesting that fatigue may have a more limited impact on sprinting ability.

Beyond contextual factors such as pacing strategy, effective playing time or match outcome, our findings highlight a clear link between match-related performance decline and aerobic fitness. Players with higher aerobic fitness, reflected by higher sLTs and PIS, showed smaller declines in both ACC/DEC actions and running activity.

**Practical Applications**

This study highlights that aerobic fitness is a key determinant for maintaining match-related physical performance, particularly TD, HIR, VHSR, and VHIR, as well as the ability to perform ACCs and DECs. Accordingly, strength and conditioning staff should implement endurance training methodologies that enhance LTs/VTs (26), PIS, or V̇o_2_max using continuous endurance training, HIIT, and sprint interval training (34). Although jump performance was not associated with performance decline, the development of strength and power capacities remains essential for overall football performance (49). Practitioners should continue to optimize the force–velocity–power profile to enhance MSS in straight line and with change of direction, as well as jump performance (51). Beyond training prescription, aerobic fitness may also support more effective in-game team management (e.g., strategies and substitutions). The marked decline in several physical performance indicators from P5 onward (60th minute) suggests that players with lower aerobic fitness levels should ideally be substituted between the 60th and 75th minute. This is consistent with evidence showing that substitutes cover 15–60% more distance than starters (56). Therefore, earlier (∼60th–75th min) rather than late changes may be more effective for sustaining team intensity toward the end of matches and influencing match outcomes (37,55). However, owing to physical demand differences between positions, these recommendations should be adapted according to the player's role on the pitch.

## Supplementary Material
